# Supplementary material for: A set of multi-entry identification keys to African frugivorous flies (Diptera, Tephritidae)
Source: Zookeys. 2014 Jul 24;(428):97–108. doi: 10.3897/zookeys.428.7366 (PMC4143993; doi:10.3897/zookeys.428.7366)
Supplement: Supplementary material 4 — Key to Capparimyia [file zookeys-428-097-s004.zip › SF4_ZooKeys_key to Capparimyia/lucid_player/help/help/numeric_features.htm]

Choosing numeric features


# Choosing numeric features

For numeric features, you must enter a value rather than choose from
a set of states. Length and width features, for example, may be treated
in Lucid keys as numeric rather than multi-state features.

Numeric features are preceded by an edit box rather than a checkbox:

To enter details about a numeric feature, click its edit box. A dialog
box will pop up into which you can type the appropriate value.

You can enter single values and ranges. Valid entries will be of the
form:

- x (a single value)
- x-y (between x and y)
- <x (less than x)
- >y (greater than y)

It's good practice when using numeric features to enter a range rather
than a single value if possible. For example, if a key to plants has a
feature *Leaf length*, it's better
to choose several typical leaves from your specimen and to enter the range
of leaf lengths found over those leaves (ignoring unusually small or unusually
large leaves) than to enter the length of a single leaf.

**Note** that the Lucid3
Player assumes an accuracy for numeric values based on the number of decimal
places specified. For example, entering the value 10 (no decimal places)
will be read by Lucid3 as meaning the range between 9.5 and 10.5; entering
10.5 will be read as the range between 10.45 and 10.55. Hence, if you
enter the single value 10, an entity scored as having the value 9.6 will
be regarded as a match.
